# Lightweight Distributed Provenance Model for Complex Real–world Environments

**DOI:** 10.1038/s41597-022-01537-6

**Published:** 2022-08-17

**Authors:** Rudolf Wittner, Cecilia Mascia, Matej Gallo, Francesca Frexia, Heimo Müller, Markus Plass, Jörg Geiger, Petr Holub

**Affiliations:** 1 BBMRI-ERIC, Neue Stiftingtalstrasse 2, 8010 Graz, Austria; 2 Faculty of Informatics, Masaryk University, Botanická 68a, 602 00 Brno, Czech Republic; 3 Institute of Computer Science, Masaryk University, Šumavská 416/15, 602 00 Brno, Czech Republic; 4 CRS4 – Center for Advanced Studies, Research and Development in Sardinia, Loc. Piscina Manna, 09050 Pula, CA Italy; 5 Diagnostic and Research Center for Molecular BioMedicine, Diagnostic & Research Institute of Pathology, Medical University of Graz, Neue Stiftingtalstrasse 2, 8010 Graz, Austria; 6 Interdisciplinary Bank of Biomaterials and Data Würzburg (ibdw), University and University Hospital of Würzburg, 97080 Würzburg, Germany

**Keywords:** Biotechnology, Technology, Medical research, Computational biology and bioinformatics

## Abstract

Provenance is information describing the lineage of an object, such as a dataset or biological material. Since these objects can be passed between organizations, each organization can document only parts of the objects life cycle. As a result, interconnection of distributed provenance parts forms distributed provenance chains. Dependant on the actual provenance content, complete provenance chains can provide traceability and contribute to reproducibility and FAIRness of research objects. In this paper, we define a lightweight provenance model based on W3C PROV that enables generation of distributed provenance chains in complex, multi-organizational environments. The application of the model is demonstrated with a use case spanning several steps of a real-world research pipeline — starting with the acquisition of a specimen, its processing and storage, histological examination, and the generation/collection of associated data (images, annotations, clinical data), ending with training an AI model for the detection of tumor in the images. The proposed model has become an open conceptual foundation of the currently developed ISO 23494 standard on provenance for biotechnology domain.

## Introduction

The exchange of research data and physical specimens has become an issue of major importance for modern research. At the same time, many reports indicate problems with quality, trustworthiness, and reproducibility of research results, mainly due to poor documentation of data generation or the collection of biological or environmental specimens serving as a source of the data^1–7^. The significant impact of flawed research results on health, economics and political decisions has been frequently stated^8–11^. Consequently, professional societies and research initiatives call for improved and standardized documentation of data and specimens used in research studies^12–17^.

**Fig. 1:**
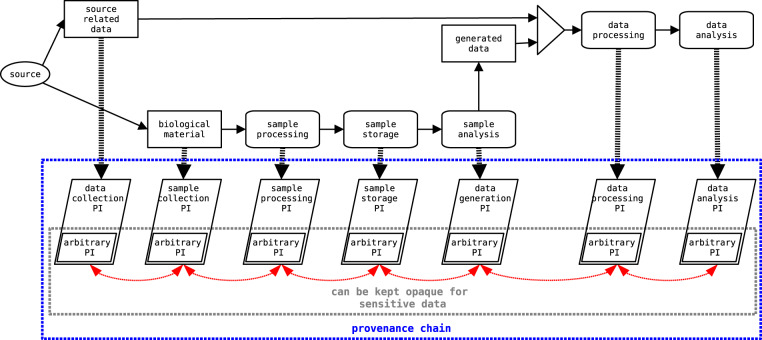
Distributed provenance chain scheme.

Provenance information documents the lineage and processing of an object^18^, such as research data or biological or environmental samples. The state of an object depends on previous activities and conditions that led to or influenced the object’s creation. If the provenance is to be used as documentation of a research object and its development, it must be linked to the provenance of its precursors, thus documenting the conditions and impact of preceding processing. By applying this approach iteratively, the provenance is forming a chain of provenance, the components of which may be distributed across different organizations and individuals that contributed to the research object and its predecessors (Fig. 1).

In order to maximize the benefits of the resulting provenance chain, each individual component of the chain should be created in due consideration of the future integration with provenance information coming from most different sources^18^. An essential aspect of fully functional machine-actionable provenance chains are interconnections which enable traversal through it, regardless of the number of intermediate steps and the originator of the particular piece of provenance. These interconnections must be fault-tolerant to discontinuities in the provenance chain, for instance, if a part of the chain is missing. The creation of such distributed provenance chains requires a consistent framework, prescribing the compatible interconnection of distributed provenance parts. In addition, a plain and coherent framework is required to facilitate the adoption of provenance by relevant stakeholders. Integration of provenance coming from different sources also demands for additional general requirements for the underlying provenance model and distributed provenance management. In particular, functional integration requires provenance syntactic and semantic interoperability^18^, advanced access control^18,19^, and a sufficient level of trustworthiness achieved by maintaining the integrity of each component of the chain^19^ and of the chain as a whole. As it will be shown in this publication, a provenance model that would enable the creation of distributed provenance chains, and provide support for the additional requirements, does not exist.

Here, we introduce a lightweight provenance model which supports the creation of distributed provenance chains in a complex, multi-organizational environment. The chain can be traversed using a single algorithm, regardless of the particular process documented in provenance since domain-specific provenance information is only attached to the chain. In addition, the provenance model can deal with situations where parts of the distributed provenance chain are missing. The provenance model, together with the corresponding requirements and recommendations, provides the prerequisites for maintaining provenance integrity and non-repudiation through the immutability and versioning of all components of the chain. The main features of the proposed model are its simplicity and ability to handle a wide range of complex real-world scenarios. It is domain- and technology-agnostic and designed to be adopted in different areas of research. It also supports the creation of provenance information for both, digital objects (such as research data) and physical objects (such as biological samples or organisms) and links physical and associated digital objects to a common provenance chain. The feasibility of the proposed provenance model is demonstrated on a real-world use case.

A complete chain of distributed and interconnected provenance components may bring important benefits if applied in research, especially in life sciences, being our main focus. Firstly, a distributed provenance chain enables the traceability of objects used in research by providing links to its precursors or successors. By this the aforementioned reproducibility issues are addressed since reproducing a process implies tracing of its inputs and outputs. Depending on the exact content of domain-specific provenance, a distributed provenance chain could be used to address many other features – for example, effective assessment of quality and fitness-for-purpose of data and their origins, tracing error propagation across a distributed research chain, or tracing back the donors of biological material in case of an incidental finding regarding their health condition, resulting from processing and analysis of a dataset. In the context of legal requirements, enhanced traceability of objects would support tracking the withdrawal of a donor’s consent, or traceability of permits required for biological material collection (e.g., w.r.t. Nagoya protocol (https://www.cbd.int/abs/)).

The model has been developed and piloted on the real-world research pipeline coming from the digital pathology domain. The proposed provenance model forms a conceptual foundation of a provenance standard for life sciences developed in the International Organization for Standardization (ISO) Technical Committee “Biotechnology” ISO/TC 276, registered as project ISO 23494 in the working group 5 “Data processing and Integration” as ISO 23494 series^20^.

### Running example

The use case, which serves as a running example throughout this publication and was used in the development of the provenance model, comes from the digital pathology domain. Digital pathology is a research field applying achievements in imaging technologies and machine learning to develop systems capable of diagnosing or supporting diagnosis of patients, based on their clinical data and high-resolution scans of histopathological biological material (so-called Whole Slide Images, WSIs). The goal of the use case is to train AI models to detect the presence of tumorous cells in whole slide images of human prostate tissue sections. The use case was chosen for its complexity, demonstrating various aspects of distributed provenance information. In particular, it requires documentation of both physical and digital objects and spans a range of diverse processes and organizations.

The use case consists of six major consecutive steps:

1. **Biological sample acquisition** (Step 1) is done in a clinical environment. Primary samples are collected during a clinical intervention – prostate biopsy – and sent to pathology for examination.
2. **Sample pre-analytics, diagnostics, and data generation** (Step 2) are done as part of the examination process – the diagnostics. The examination consists of the gross evaluation, generation of tissue blocks, cutting of tissue blocks into slides, staining and scanning the slides. A set of annotated scans – Whole Slide Images (WSIs) – together with additional information is used for diagnosis. The annotation describes tumor areas and morphological features, such as the Gleason score in the case of a prostate biopsy.
3. **Biobanking** (Step 3a) is performed after the diagnosis is finalized. The samples are stored in a controlled environment and provided through a searchable database for future use. Thereby a defined cohort or set of samples can be retrieved to address specific research questions.
4. **WSI preprocessing** (Step 3b) prepares the WSIs to be processed by a computational workflow for machine learning. Each WSI is assigned either to the training or test dataset. Since the workflow is not capable of processing high-resolution WSIs, the images in the datasets are split into smaller patches, filtered, and labeled. The indices of unfiltered patches are saved to disk.
5. **AI model training** (Step 4). The AI model training iterates in two steps: model training and model validation. In both steps, the model generates predictions for labeled pairs of patches extracted from the WSIs using the index file. The model parameters are updated during the training step only. During the validation step, a portion of the dataset reserved exclusively for validation is used to assess the quality of the model at regular intervals. Validation is also used to determine whether any hyperparameters (parameters of the learning algorithm) need to be adjusted. The result of this step is a trained model.
6. **AI model testing** (Step 5). The trained model is applied to the unprocessed test dataset. The results are evaluated using both automated metrics as well as manual inspection by an experienced pathologist. This step is done in order to assess the overall performance of the pipeline.

The use case serves as a running example throughout this paper to demonstrate how the proposed provenance model can be used to capture required semantics. How the proposed provenance model is used to design a provenance chain spanning all steps of the example is described in detail in the attached supplementary material. Automated provenance generation based on existing logs was implemented for the computational steps of the use case (steps 3b, 4, and 5).

## Results

### Novelty & contributions

The aim and the main contribution of this work is a lightweight provenance model to create distributed provenance chains. The heterogeneity of the research environments is addressed by enabling the generation of domain-specific provenance by different organizations for their processes and attaching it to the provenance chain with a pre-defined structure. Additional requirements facilitate integrity and non-repudiation of the arbitrary components by their immutability property and enable a method for preventing provenance information deprecation. In particular, the main contributions of our work are as follows.

1. Introduction of a **provenance backbone**, as part of a provenance bundle – individual component of a provenance chain – which is required for the navigation through distributed provenance chains. The backbone is designed as a standardized expression of derivation paths between inputs and outputs of a documented process in a provenance graph.
2. Proposal of how to deal with **missing provenance components** in a provenance chain.
3. Proposal of **requirements related to distributed provenance management**, such as provenance versioning, integration and interpretation of persistent identifiers in provenance, and adding support for immutable provenance records, which is a prerequisite for integrity and non-repudiation of provenance.
4. Demonstration of **applicability, usability, and benefits of the backbone** using the running example. The running example concerns the evolution of an AI model trained to support pathologists in cancer diagnostics. The running example covers the whole process pipeline starting from biological material acquisition, through processing, data generation, and, finally, data processing by the AI model.

In this section, we define individual parts of the provenance model and show how the model is applied to the respective parts of the running example. The application of the proposed model to build up a provenance chain is shown, explained, and implemented in the supplementary material (see the Data and Code Availability sections for references).

Our work is built on top of the PROV^21^ provenance model, provenance composition pattern^22^ and compound activities pattern^23^. We refer readers who are not familiar with these concepts to the Methods section, where we also describe steps that lead to the design of the proposed model and future developments.

We would like to point out that integrity and non-repudiation of the chain, prevention from provenance information deprecation, and support for opaque provenance components are partially out of the scope of this publication. The related mechanisms to address these requirements, which are briefly described in the Results section, should demonstrate that the provenance backbone model was designed with these additional requirements in mind, but detailed explanations and demonstration of the concepts are subject to separate publications.

A preliminary version of the proposed provenance model, including aspects related to distributed provenance management, connectors, and resolving shared identifiers, has already been published as an EOSC-Life project (https://www.eosc-life.eu) deliverable^24^.

### Related work

The related work focuses primarily on current options for constructing distributed provenance chains and their representation. The first part focuses on the standardized expression of derivation paths between inputs and outputs of a documented process in provenance. The remaining part deals with methods that can be used to interconnect provenance bundles coming from different sources.

The common way to express provenance is a graph with annotated nodes and edges^25,26^. Nodes in the graph typically represent documented processes or objects, and edges represent their relations. The annotations are used to attach semantics to nodes and edges. The PROV model as devised by the World Wide Web Consortium (W3C) is a family of specifications^21^ defining a widely-accepted standard, which can be regarded state-of-the-art for provenance information representation. The standard intentionally defines a general provenance information model (see the Methods section for further details about the model) so that it can be adapted to all kinds of domains^18,27–38^. The universality is a desired property because adoption of PROV does not introduce significant constraints or necessitates additional requirements for specific solutions. On the other hand, since centralized management is not envisioned, the adoption of the PROV standard often leads to incomplete, incompatible, and inconsistent solutions^39,40^. Existing provenance solutions typically utilize arbitrary derivation paths between inputs and outputs of a process, which are not intended to be generalized and adopted by other domains – one of the main gaps we are addressing in this work. With arbitrary derivation paths, traversal through such provenance chains in the sense of locating all inputs that affected the given output of a process (or vice versa), using a single algorithm for different graphs, would be possible only by applying a general graph search algorithm, such as Depth-first search or Breadth-first search^41^. This is an issue since provenance graph can be multiple times bigger than documented data^42^ leading to unnecessary complexity when applying general algorithms. If an arbitrary derivation path contained sensitive information without any constraint, a general traversal algorithm would generally need to have access to it to be able to reach components of the graph which are separated by sensitive information. The derivation paths proposed in our model are designed in a way so that they do not contain sensitive information, and the traversal algorithm does not need access to it, conforming to the least privilege principle^43^.

In order to create a provenance chain, provenance bundles must be interconnected. One of the most intuitive methods to interconnect standalone provenance parts is based on the idea of shared identifiers, which has been already used in several implementations^27,44^. For example, it has been recommended as a best practice to create links between provenance bundles documenting different granularity of computational workflow provenance^27^, or it was used in^44^ to create a so-called whole-network provenance to capture provenance in an operating system. The core concept is based on an identifier shared by different provenance graphs. Once those graphs are merged, they will be pasted along the node with the shared identifier (this operation works similar to the *join* operation in relational databases). The concept of the idea of shared identifiers in the sense of creation of linking provenance graphs was formally defined in the provenance substitution pattern in 2016^45^. The referenced work defines a formal framework for the substitution of provenance information with more detailed provenance information, which is an inverse function of provenance abstraction^46^. Provenance composition pattern^22^ builds on top of the provenance substitution by introducing a minimal solution for composing provenance in PROV. On the other hand, as also stated by the authors of the original work, the pattern does not address practical aspects of distributed provenance, such as conventions for sharing identifiers or normative definitions of attributes containing information to navigate provenance bundles. In addition, the pattern also suggests updating the sender’s provenance information to enable forward navigation by adding additional information. This approach, however, renders impossible, for example if the sender’s provenance information has already been digitally signed, and an update of the original provenance information would invalidate the signature, by this affecting the integrity and trustworthiness of the whole provenance chain. These gaps are addressed in the present work by the concept of shared identifiers and refinement of the provenance substitution pattern to express links between distributed provenance bundles. We have also provided requirements related to practical issues, such as generating and sharing identifiers, which are not addressed in the original work. The provenance composition pattern is described in more detail in the Methods section.

A different way of linking standalone provenance bundles is suggested in the aforementioned PROV standard^47^ providing a method to map nodes in provenance graphs with different identifiers. While the mechanism is useful in some situations, it is not appropriate to be used as a base mechanism for creation of distributed provenance chains. According to the PROV validity constraints^48^ applying the method to create bi-directional links between two provenance bundles directly would lead to invalid provenance instances. A detailed explanation of this problem is provided in^49^.

### Provenance finalization event

The main goal of provenance information is not to include or replace existing documentation or logging infrastructure but rather to provide an additional level of documentation, which should be fully technically interoperable across different organizations and potentially fulfilling additional requirements (e.g., recommendations listed in^18^). The reason is that existing logging infrastructure serves primarily for resolving technical or security incidents, while provenance information provides long-term machine-actionable documentation of processes.

This can be achieved by assembling the required information in the conventional format and translating it into appropriate provenance representation when necessary: on request, at the end of the specified process, or periodically after a pre-defined time interval. Specific examples could be when requesting biological samples from a biobank, requesting imaging data for further processing, or at the end of the AI model training. This instant of time is referred to as *provenance finalization event*.

To maintain consistency and coherence, any provenance information created during the finalization event has to be a **valid PROV instance encapsulated in a PROV bundle** containing all relevant entities, activities, and agents documenting domain-specific information. Its content is considered to be a **fixed and immutable snapshot of the current knowledge** and is not further directly updated. Such provenance information can be normalized, digitally signed, and archived to be used for auditability, accounting, or other purposes. This allows the generation of valid and fairly complete provenance information encapsulated in a bundle documenting a particular process. From this point, we use the term provenance information to refer to the provenance information generated during the finalization event.

By applying this mechanism, the resulting provenance chain documenting a distributed process (such as the research pipeline presented in the running example) is technically formed by interconnected provenance bundles, where each bundle documents a separate part of the process, and can be managed independently. As a result, the provenance chain documenting the running example consists of six bundles (Fig. 9). In order to build up corresponding provenance chain, the interconnections between the bundles must be created. In this regard, several aspects must be taken into account before usage of the proposed provenance model: a) determination of the number and meaning of the interconnections between different bundles; b) granularity level of provenance description; c) level of collaboration between organizations generating interconnected provenance bundles. These three aspects are elaborated on in more detail in the Discussion section after the proposed provenance model is introduced.

### Provenance backbone

**Fig. 2:**
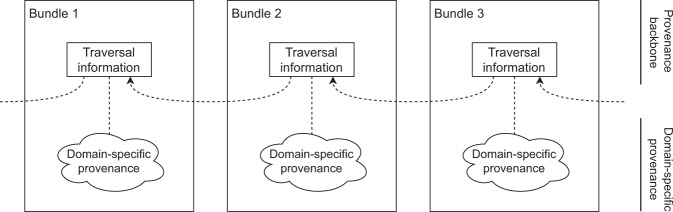
Two types of semantics in a distributed provenance chain.

The most crucial observation related to our contribution concerns semantics included in a distributed provenance chain. In order to enable traversal through provenance chains, each provenance bundle included in a chain must contain information that will enable the traversal. In this context, the semantics of contents of a provenance bundle in a provenance chain can be divided into the two following categories (Fig. 2):

1. **Domain specific information** documenting details of executed process. This type of information is included in any bundle in the distributed provenance chain, and its content is always dependent on the type of process being documented (e.g., biological material acquisition, sample processing and storage, data generation and processing).
2. **Traversal information** required to traverse the distributed provenance chain. This type of information is included in any bundle in the distributed provenance chain but is not affected by documented type of process.

The *Provenance backbone* is part of a distributed provenance chain formed by the traversal information required for navigation through the chain and to which the domain-specific provenance information can be attached. The expression of backbone in a bundle has a prescribed structure, which can be seen as a dependency pattern^50^ to express the traversal information. The backbone is built on top of connectors and harmonized expression of derivation paths between documented inputs and outputs in individual provenance bundles.

### The connector

*Connector* is a provenance structure (*prov:entity*) with a shared identifier that represents an object that is exchanged between two organizations – a sender and a receiver. Examples of exchanged objects are biological samples (between step 1 and step 2) or a dataset (between step 2 and step 3). The connector represents a snapshot of the exchanged object at the time of its sending and should be present in both the senders’ and receivers’ finalised provenance. To distinguish between connectors representing inputs and outputs, the entity expressing the connector as output is attributed *senderConnector* type. The entity expressing the connector as input is attributed *receiverConnector* type. Attributes of the connector are used to store information required to enable traversal between provenance bundles. In particular, the connector contains at least the following information about a destination bundle (destination bundle in the sense of the opposite side from the perspective of a particular organization, which is the subsequent bundle for the sender and the preceding bundle for the receiver):

- identifier of a destination bundle – a preceding or subsequent bundle in the provenance chain. The destination bundle contains provenance documenting preceding/subsequent process.
- URL of a service, where the content of destination bundle can be requested.

The connector can also contain additional technical information related to locating and accessing the remote bundle (see^51,52^). Technical details about the format of the shared identifier of the connector and about preventing from collisions between identifiers of other provenance structures are available in our EOSC-Life project deliverable^24^.

**Fig. 3:**
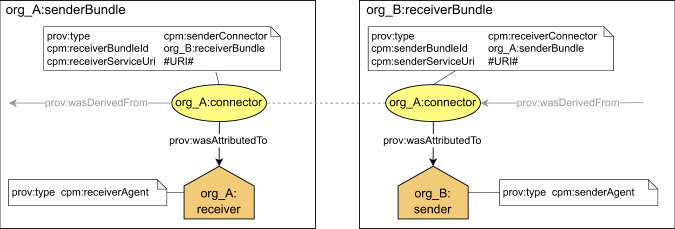
Detailed schema of connectors and their usage in provenance information. Since identifier of the connector is shared between the sender and the receiver, it must have the same prefix, which is the prefix identifying the sender of the described object.

A schematic view of the connectors is depicted in Fig. 3. According to the use case example, the exchange of WSI data between a hospital pathology department, where the data was generated during the optical microscopy process (step 2), and a data science institute, where the data is used as an input for further processing (step 3), the connectors are used as follows. The data are represented in the hospital’s and the institute’s provenance information as a connector with a shared identifier. In the provenance of the hospital, the connector would be attributed the *senderConnector* type. In provenance of the data science institute, the connector would be attributed the *receiverConnector* type. By applying the proposed mechanism, each connector can refer to at most one bundle.

### Harmonized derivation paths

**Fig. 4:**
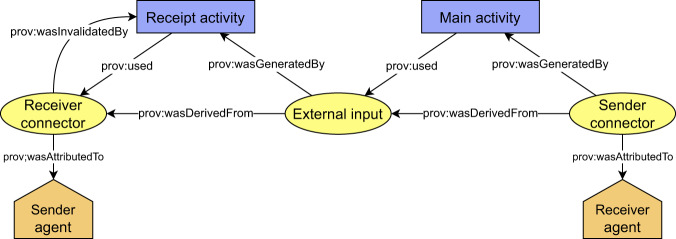
Conceptual schema of a piece of the provenance backbone, which is present in a single provenance bundle.

Building on the top of the connectors, we continue with the definition of harmonized representation of derivation paths. The derivation paths document how outputs of a documented process were derived from their inputs. Technically, the provenance backbone is formed by prescribed form of derivation paths between *senderConnectors* and *receiverConnectors*. In addition to connectors, the derivation paths contain the following types of provenance structures (Fig. 4):

- An *externalInput* entity that represents the exchanged object at the time of its receipt by the receiver. The difference between the *receiverConnector* and *externalInput* is that the *receiverConnector* represents a snapshot of the exchanged object at the time of its sending, while the *externalInput* represents a snapshot of the exchanged object at the time of its receival.
- A *receiptActivity* activity that represents how output of the previous process (*receiverConnector*) became an input for the current process (*externalInput*).
- A *mainActivity* activity that represents how input of the current process (*externalInput*) became an output of the current process (*senderConnector*).
- *SenderAgent* and *receiverAgent* structures that represent responsible organizations for preceding/consecutive steps in the chain.

**Fig. 5:**
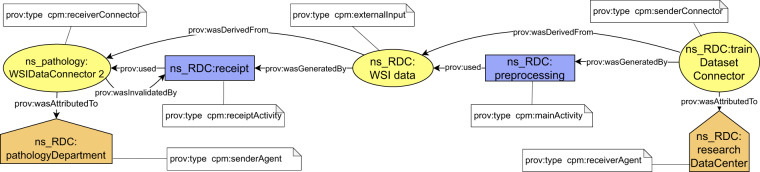
Schema of provenance backbone applied to WSI data preprocessign step (step 3b).

Considering the WSI preprocessing step of the running example (step 3b), the *receiverConnector*, *externalInput* and *senderConnector* entities would represent the dataset at different time instances. The *receiverConnector* represents the state of the input dataset at the moment of its sending from the pathology department (a sender), and the *externalInput* represents the state at the moment of its receipt by the data science institute (a receiver), and the *senderConnectors* represents the state at the moment of sending the dataset to a consecutive step – AI model training (step 4). The *receiptActivity* represents how the dataset was received, and the *mainActivity* represents dataset preprocessing process. The corresponding part of the provenance backbone is depicted in Fig. 5.

Once all the required types of provenance structures forming the provenance backbone are defined, the definition of the backbone is completed by specifying how the structures are mutually interconnected to form derivation paths (general schema was depicted in Fig. 4 and its application in Fig. 5):

- The *receiptActivity* activity uses (*prov:used*) *receiverConnector* entities and generates (*prov:wasGeneratedBy*) an *externalInput* entities.
- The *receiptActivity* activity invalidates (*prov:wasInvalidatedBy*) the *receiverConnector* entities.
- The *mainActivity* activity uses (*prov:used*) the *externalInput* entities and generates (*prov:wasGeneratedBy*) *senderConnector* entities. Each pair of *externalInput* and respective *senderConnector* is related using derivation (*prov:wasDerivedFrom*) relation if and only if the particular *senderConnector* was somehow affected by the *externalInput*.
- The agent entities are attributed (*prov:wasAttributedTo*) to the connectors.

**Fig. 6:**
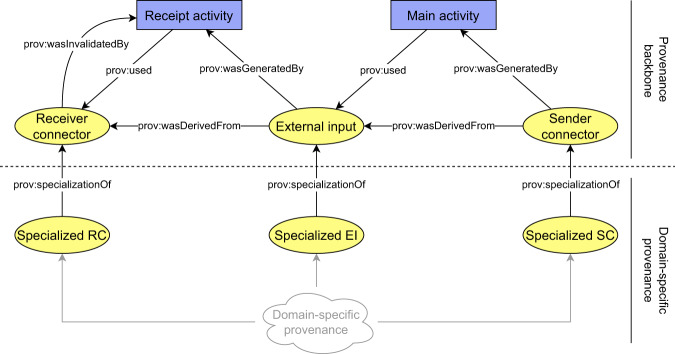
Conceptual schema of attachment of domain specific provenance to provenance backbone. The domain specific provenance is related to the backbone by applying the compound activities pattern. The *receiverConnector* (RC), the *externalInput* (EI) and the *senderConnector* (SC) types are included only in the backbone. Agents are omitted for simplicity.

In general, there may be multiple *receiverConnector*, *externalInput* and *senderConnector* entities present on a backbone to express multiple inputs and outputs of a process. The backbone always contains at most one receipt activity for each pair of *receiverConnector* - *externalInput* and at most one *main activity*. Table 1 summarize the semantics of the backbone in cases when some of the defined entities are missing. Any other domain-specific information should be expressed outside provenance backbone and attached to the backbone using the compound activities pattern. The general schema of a domain-specific information attachment is depicted in Fig. 6.

**Table 1:** Semantics of the provenance backbone with missing entities.

	Entities in the backbone	Missing entities	Description of semantics	Figure
1.	*senderConnector*	*receiverConnector externalInput*	Beginning of a provenance chain. The process does not have any external input coming from a previous process.	Fig. 10a
2.	*senderConnector externalInput*	*receiverConnector*	Documented process had an external input, but the information where to find its provenance is not available.	Fig. 10b
3.	*receiverConnector externalInput*	*senderConnector*	End of a provenance chain. Documented process does not produce any outputs that have been sent to a consecutive process.	Fig. 10c
4.	*externalInput*	*receiverConnector senderConnector*	End of a provenance chain. Previous provenance of an external input can not be found and the documented process does not produce any outputs that have been sent to a consecutive process.	Fig. 10d

**Fig. 7:**
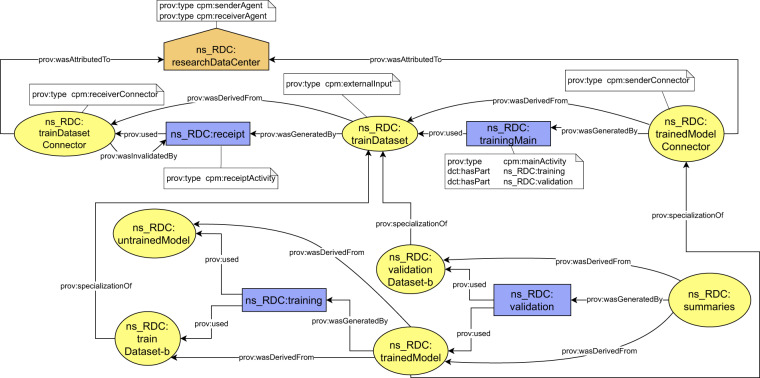
Schema of domain specific provenance attachment applied to AI model training step (step 4).

Considering the AI model training step (step 4) of the running example, the domain-specific provenance may include further details about how the AI model training was performed. In our implementation, it contains hashes of WSI data files and references to their location; a reference to git repository including implementation of the initial untrained AI model; a representation of AI model configuration used for training; and further details about each step of the training and continuous validation process (*epochs* in the AI terminology). An example of the domain-specific provenance attachment to provenance backbone is depicted in Fig. 7. We refer readers to the Data and Code Availability sections for a practical example of how we expressed and implemented automated provenance generation for real-world AI workflow.

**Fig. 8:**
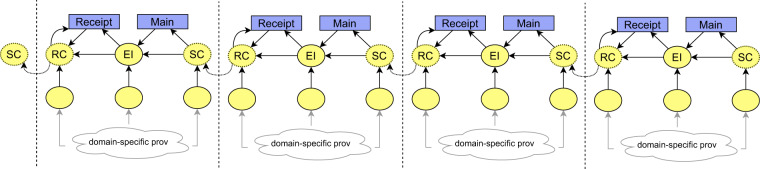
Conceptual schema of how the provenance backbone forms a distributed provenance chain. Each element of the chain is generated by different organization and is interconnected with other elements using the connectors with shared identifiers. Agents are omitted for simplicity.

**Fig. 9:**
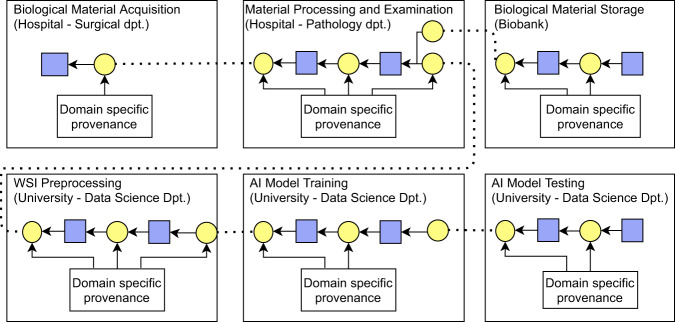
Simplified schema of a distributed provenance chain for the running example.

The general schema of the resulting provenance chain based on the provenance backbone is depicted in Fig. 8, and its adoption for the running example is depicted in Fig. 9.

**Fig. 10:**
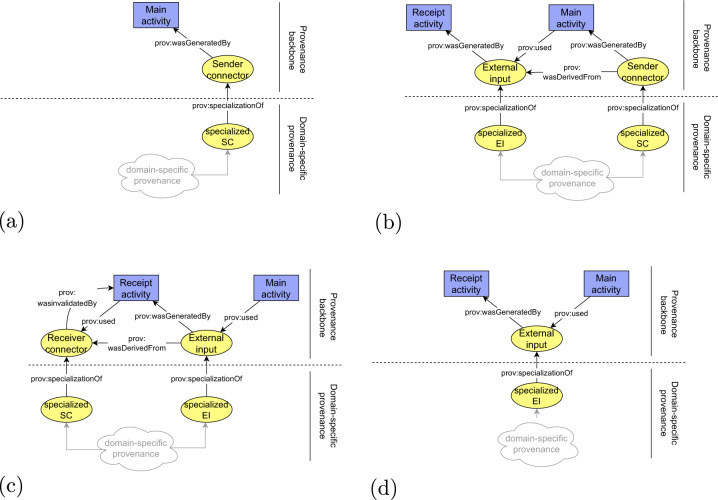
Conceptual schema of the provenance backbone with missing connectors. Agents are omitted for simplicity. **(a)** The provenance backbone without receiver connector and external input. **(b)** The provenance backbone without receiver connector. **(c)** The provenance backbone without sender connector. **(d)** The provenance backbone without connectors.

As a result, a query “Select all traceable inputs for a given output” can be algorithmically solved by applying a graph search algorithm starting from the *senderConnector* and finding all directed paths of the length two with edges of type *prov:wasDerivedFrom*. By applying this, the resulting time complexity of the proposed algorithm for traversal is bounded by the number of traceable inputs and outputs documented in a provenance bundle. In contrast, this is not the case with arbitrary derivation paths since they can generally be of arbitrary length. Similarly, the algorithm can be run in an analogous way to get all outputs affected by the specified input. After the algorithm finds a connector, it can use the URIs stored within its attributes to query for previous or consecutive provenance in the chain. If the result of the query is a provenance bundle containing the backbone, then the same algorithm for finding inputs or outputs can be applied to navigate to another bundle, giving us **a general algorithm for navigation through distributed provenance chains**.

#### Missing provenance components

**Fig. 11:**
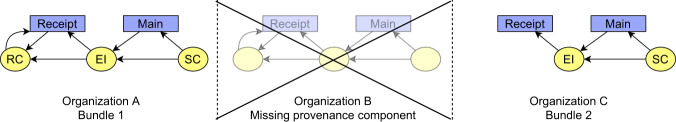
Conceptual schema of the provenance backbone with a missing provenance component. In terms of connectors, the Bundle 1 contains a *senderConnector* to express that the particular described object has been sent to another organization. The *senderConnector* does not contain values of its attributes (especially the consecutive bundle identifier and service URL needed to navigation), so technical information to navigate through distributed provenance is missing. On the other hand, the Bundle 2 does not contain *receiverConnector*, because the state of particular described objects in the time of its sending from the sender is not known to the receiver. The Bundle 2 contains *externalInput* entity to express that the described process has an external input.

In a realistic scenario, we can not presume that every organization will generate provenance information for its activities. For that reason, there must be a mechanism to deal with missing provenance components in the chain so that a general algorithm for navigation through distributed provenance chains will not be disrupted. A general schema of a backbone with missing provenance components is depicted in Fig. 11.

There are three organizations depicted in the figure: organization A, organization B, and organization C. Presume that the corresponding provenance information that would be generated by Organization B does not exist. In such a case, it is desired that the navigation from provenance information of Organization A to Organization C (or vice versa) is still possible.

**Fig. 12:**
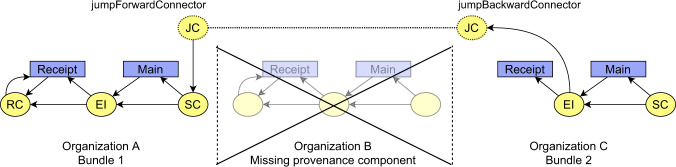
Conceptual schema of the provenance backbone with a missing provenance component including *jumpConnectors*.

To prevent from breaking of the provenance chain, the bundles generated by A and C should be interconnected. To achieve this, we propose using another two types of connectors: *jumpForwardConnector* and *jumpBackwardConnector*. Similarly to *receiverConnector* and *senderConnector*, the jump connectors are entities with shared identifiers, which are related to particular *externalInput* or *senderConnector* entities, and which technically implement the interconnections between distributed bundles. In contrast to *receiverConnector* and *senderConnector*, the semantics of jump connectors is different. The jump connectors do not represent an exchanged object simply because there is no object exchanged directly between the two processes. *jumpForwardConnector* expresses an output of a described process, whose derivatives are used in a non-adjacent consecutive step. *jumpBackwardConnector* expresses an object from which an input of the described process was derived and which is coming from a non-adjacent previous step. To express this in the proposed model, *externalInput* entity is derived (*prov:wasDerivedFrom*) from *jumpbackwardConnector* and *jumpForwardConnector* entity is derived from *senderConnector*. Attachment of domain-specific provenance to the backbone is thus not affected by the presence of the jump connectors. The general schema of the backbone, including the jump connectors, is depicted in Fig. 12.

Attributes of the jump connectors are then used to store location information about destination bundles:

- identifier of a destination bundle - the bundle which contains provenance information documenting a non-adjacent previous or consecutive step.
- URL of a service, where the content of the destination bundle can be requested.
- identifier of an entity (*externalInput* or *senderConnector*), to which the jump connector is related in the destination bundle.

**Fig. 13:**
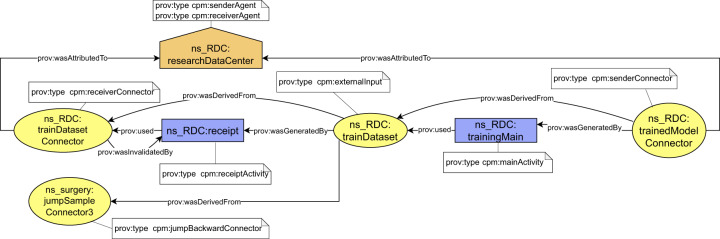
Schema of *jumpBackwardConnetor* usage in the AI model training step (step 4) provenance to refer to biological sample acquisition bundle (step 1).

Usage and the benefits of the *jumpConnectors* are demonstrated for the AI model training step (step 5) of the running example. An exemplary goal would be to confirm that the training dataset used as an input is not fabricated and is coming from real patients. This might be accomplished starting from the AI model training bundle, where the *externalInput* entity representing the dataset would be derived from a *jumpBackwardConnector* representing the acquired sample in the first step of the chain – biological material acquisition provenance generated by the hospital (step 1). Provenance backbone for step 5, including the corresponding jump connector, is depicted in Fig. 13.

Depending on whether a particular user executing the query would have access to the hospital’s provenance bundle, two situations can occur. First, access is granted, and the user can inspect the content of the bundle to verify the required information. Second, access is not granted. On the other hand, despite not having direct access to the full content of the bundle, the successful verification that such a provenance exists and is coming from a reliable institution might be a good indicator of not fabricated dataset (unlike in a COVID-19-related Surgisphere case^2^). If a provenance coming from a hospital could not be found, or its existence could not be confirmed, one can have doubts regarding the original source of the data. Using the backbone without the jump connectors, this verification would be feasible only if a complete chain exists to enable step-be-step traversal. A drawback of not having the jump connectors is that the algorithm traversing the distributed provenance chain would need to traverse through parts of distributed provenance, which may not be of primary interest.

### Preventing from provenance information deprecation

Provenance information contains a description of an object, which might have been deprecated over time. At the same time, directly updating provenance information to reflect the current state of described objects might be not appropriate or feasible—e.g., because provenance generated during the finalization event is already archived and may be digitally signed. We propose two mechanisms to deal with the deprecation of provenance information without its direct updating. The first mechanism relies on our proposal for bundles versioning using a meta-bundle, and the second mechanism relies on globally unique and resolvable persistent identifiers (PIDs)^53^. The more technical description is present in our EOSC-Life project deliverable^24^. A more detailed description of the bundles versioning mechanism is shown in the supplementary material.

#### Bundles versioning

First, every organization involved in distributed provenance chain maintains a meta-bundle that represents meta-information about finalized bundles of a particular organization. In particular, every finalized bundle is represented in the meta-bundle as an entity. To update a bundle, a new copy of the bundle is created. The new bundle is perceived as a replacement of the original bundle and includes any modification of the original bundle - additional, removed, or updated provenance information. We suggest that the original bundle is not deleted since this would disrupt the original provenance chain containing the references to the old bundle. The update is expressed in the meta bundle by applying a revision pattern^23^ loosely following semantics defined in the PAV ontology^54^.

**Fig. 14:**
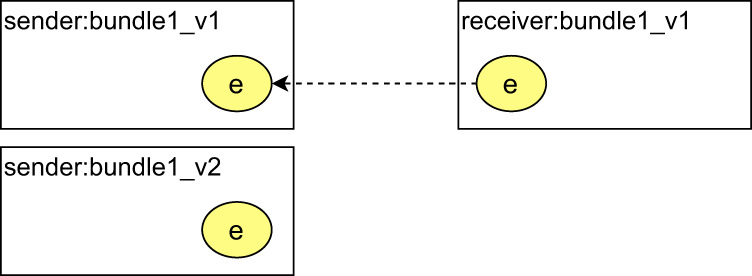
Connection between two bundles created via *connector*, which is not affected by updating the bundle. This enables sender to create hashes and digital signatures of a bundle and to provide them to a receiver, which may include them into his provenance information to enable further integrity check.

By applying this mechanism for bundles versioning, the history of all previous versions is preserved. This property is crucial in the context of distributed provenance since any provenance bundle may be referred to from a different bundle by a connector, as depicted in the Fig. 14. If such an updated bundle is found during the navigation through distributed provenance, an algorithm executing the navigation may detect that an updated version exists and proceed accordingly. The check for updates can be done using the meta-bundle.

#### Resolving persistent identifiers

A PID is an identifier that is globally unique and long-term resolvable. Resolvability of an identifier refers to the property of being able to access identified object, its digital representation, or related information using the PID. PID is generated and assigned by a reliable third party (e.g., Internation DOI Foundation (https://www.doi.org/) or ePIC (https://www.pidconsortium.net/)), which provides assurance of required PID properties. PIDs can be expressed in provenance information using qualified names (containing a prefix and a local name). The interpretation of a PID follows the interpretation of any qualified name.

If a value referenced by a particular PID changes, a new value is assigned to the PID without modifying provenance information at a particular resolver. The ability to track the history of values for a particular PID is delegated to the PID assigning authority. Usage of persistent identifiers may be appropriate for attribute values that must always be up to date but are in the custody of a different organization than the organization generating particular piece of provenance. If the value of PID changes, the responsible organization only notifies PID resolver, and the new value is then “propagated” to every provenance information containing particular PID.

In order to prevent from provenance chains deprecation, a PID can also be used to identify each provenance bundle in provenance chain. In this case, the PID can be resolved to a provenance URI, which consequently dereferences to any provenance format selectable via a content negotiation protocol^51^. The PROV-AQ specification^51^ defines further technical details about how provenance can be located, retrieved, and queried using standard web protocols, such as HTTP, HTML, or SPARQL.

### Support for integrity, authenticity and non-repudiation

One of the main features of the proposed method for prevention from provenance deprecation is that the provenance information is treated as an immutable object and never directly updated after it is generated during the finalization event. Particular provenance can be afterward hashed or digitally signed and stored. This immutability property ensures that the bundles versioning mechanism does not break potential hashes or digital signatures of provenance, which can be used for verifying bundle integrity, authenticity, or non-repudiation. Further details about how to achieve non-repudiation of provenance information in a localized environment can be found in our previous work^55^; immutability-based design of the distributed model allows the same mechanism to be applied.

### Support for opaque provenance components

The backbone contains coarse granularity representation of traceable inputs and outputs of a described process, derivation paths between them, and technical information to previous and consecutive steps in a distributed provenance chain. We suggest that any other detail, which could be sensitive or not relevant on this level of abstraction, should be included in domain-specific provenance outside the backbone. This provides us a baseline categorization of confidentiality of information in a single bundle, which is part of distributed provenance chain:

1. Domain-specific information contains arbitrary information, including sensitive or confidential information (e.g., personal data).
2. Provenance backbone contains only traversal information and does not include sensitive or confidential information.

With this categorization, we can see that the proposed general algorithm for navigation through distributed provenance chains does not need access to any sensitive information documented in provenance chain, enforcing the least privilege principle^43^. The navigation algorithm can exploit the fact that the backbone is connected to the rest of the domain-specific provenance exclusively using *prov:specializationOf* relation.

## Discussion

The reproducibility crisis in research caused an urgent need for standardization of complete, reliable, and trustworthy documentation of the research process. The current issue is the lack of ability of provenance models to work in complex distributed multi-institutional environments, which requires their syntactic and semantic interoperability. General provenance models such as PROV^21^ or OPM^26^ provide a framework to achieve interoperability, but their adoption typically leads to incomplete, inconsistent, or incompatible solutions. This is primarily caused by the generality of the models so that they must be adopted^23^ to be able to capture domain-specific semantics. In addition, the solutions typically focus on specific domains and problems, but they don’t aim to be adopted in a wide spectrum of domains due to not enough authority of maturity.

To address this issue, we have introduced the provenance backbone – a provenance model to enable the creation of distributed provenance chains by harmonized expression of the common semantic – the traversal information. Expression of the common semantic in a harmonized (standardized) way is a standard method to document common situations in provenance. In provenance related literature, the concept is also called provenance pattern^56^, dependency path pattern^50^, or provenance recipe^23^, which can be implemented using provenance templates^56,57^. Although we call the resulting interconnected provenance parts as a (linear) chain, it can actually be forked and merged from/to different branches, following documentation of inputs and outputs coming from different sources.

In the context of the identified gap, the development of the proposed model can be seen as a common intermediate step in the process of standardized adoption of general provenance models within individual domains. In particular, the model prescribes a way how to represent minimal set of domain-agnostic information required to build uninterrupted provenance chains, to which arbitrary domain-specific information can be attached. As a result, the provenance backbone provides a baseline framework and interface for distributed provenance integration. The model was designed in a way that it can also support additional requirements such as provenance versioning, integrity verification, non-repudiation, and opaque provenance components. Addressing these advanced requirements is partially out of scope of this publication. Description of the proposed mechanism should demonstrate that the provenance backbone model was designed with these additional requirements in mind. Detailed explanation and demonstration of the provenance backbone integrated with the described mechanisms to address versioning, integrity, non-repudiation, and access control are subject to additional publications.

Our model lies at the core of the standardization of provenance information for the biotechnology domain pursued by Technical Committee 276 “Biotechnology” in International Organization for Standardization (ISO). Despite the ISO standards are typically not publicly available for free, the model has become an open conceptual foundation for Part 2 of the developed standard series 23494. Other developed parts of the standard build on the proposed provenance model to define provenance models for particular sub-domains: handling biological material (Part 3), data generation methods (Part 4), and computational workflows (Part 5). We believe that the model has a very good chance of being adopted by both academia and industry. Industrial adoption is particularly important as it is not realistic to generate in-depth provenance information without direct support in different devices such as laboratory automation systems handling biological material or data generation devices (e.g., microscopes, Whole Slide Imaging scanners, spectrometers, DNA sequencers).

Despite the simplicity of the proposed model, there are still some elements that must be decided and designed prior to its usage. First, the adoption requires determination of *external* inputs and outputs of a documented process. The objects expressed on the backbone are expected to be traceable in the provenance chain, which is dependent on the purpose of provenance collection. This is because not all objects used in research are always expected to be traceable – for example, in the case of the AI model training presented in the running example, the implementation of the model itself is not expressed on the backbone, it is expressed as an entity only in the domain-specific information. The reason is that for the purpose of the example, we did not consider tracking of the model implementation history to be relevant. The proposed provenance versioning mechanism can be used to handle the updates related to the inclusion of new traceable objects to the backbone.

The second important aspect for the adoption of the backbone is the granularity of documented process. The proposed model is generic enough to handle both coarse-grained and fine-grained provenance descriptions – for example, computational workflow run or specific iteration of a sub-workflow. Both cases can be designed with proper identification of inputs and outputs of a documented process. From the provenance backbone perspective, the only important part related to granularity is a common interpretation of the connectors – at both sender and receiver, the connectors on the interface must represent the same object.

Another related aspect is the expected level of collaboration between organizations generating interconnected provenance bundles. As the provenance backbone presumes, the identifier of a connector is always shared, and we argue that this is a realistic and achievable presumption. In the case of sharing identifier of *senderConnector* and *receiverConnector* between a sender and a receiver, the identifier can be exchanged together with the exchanged object. In addition, we can also presume that the receiver would inspect provenance of the exchanged object before its usage to assess its quality or fitness-for-purpose. If a particular connector entity will be already present in provided provenance, the identifier is exchanged during the provenance exchange. If the connector would not be present in provided provenance, the sender can always generate the identifier additionally, and include it in his part of the backbone according to the proposed method for provenance versioning. In cases when the identifier would not be shared or not included in either the senders’ or receivers’ provenance, the resulting provenance chain would not be formed due to missing interconnection (this can be corrected later using the proposed versioning mechanism). On the other hand, the provenance backbone model allows these situations and considers them valid, and are in fact required to express the first and the last steps of a provenance chain. The semantics of provenance backbone with missing provenance structures was summarized up in Table 1. In case of sharing identifiers of *jumpBackwardConnector* and *jumpForwardConnector*, the situation is more difficult. Creation of *jumpBackwardConnector* requires a sender to provide location information about its preceding bundles to a receiver, resulting in a snowballing effect – the further we progress in a chain, the more information is needed to share to provide all the “back links”. Despite the growing amount of shared information, backward linking is achievable solely through communication between sender and receiver during a described object exchange. In contrast, the creation of *jumpForwardConnector* requires additional communication between an original provider of an object and its every consecutive consumer (forward reachability problem^22^). This can be addressed by deployment of a provenance pingback services^22,51,52^.

The proposed mechanisms to prevent provenance information deprecation are built on the top of meta-bundles or PIDs. The advantage of the meta-bundle over PID is that it provides direct support for auditability and accountability of past modifications of provenance information by persisting all the previous versions and their relations without the involvement of a PID assigning authority. This way, trust in provenance bundle history can be established, e.g., by applying digital signatures to meta-bundle and its notarization to achieve non-repudiation^55^. A disadvantage of the meta-bundle method is that it poses further requirements on provenance management in provenance generating organization. In the case of PIDs, the requirement for persisting history of updates is delegated to a PID assigning authority. In this case, the establishment of trust in resulting provenance can be established only partially since all values represented by PIDs can be updated without direct change of provenance, so the change remains undetected solely using digital signatures. For that reason, the PID assigning authority must be independent, reliable, and trusted to behave in accordance with a protocol established in advance. In addition, privileges to initiate an update of a value assigned to a PID must be strictly defined, and the whole process must be auditable.

### Conclusion

The reported reproducibility and reusability problems with datasets and research results are often of complex nature; sometimes, it is one wrong step of the whole chain of processes how the data was created, or incompatibility of a sequence of steps (e.g., biological material was good for other analytical methods, but not for the particular method applied). Only when the complete trustworthy chain of provenance is available such problems can be identified. Machine-actionable and interoperable chain of provenance information can prevent problems from occurring at all, as provenance chain can be analyzed before the biological material and data are used for the next step; such fitness-for-purpose analysis is fundamental for meaningful and reproducible research. The proposed provenance model has been carefully designed to implement support for this in complex multi-institutional environments that are common in biotechnology and life sciences.

In this paper, we have proposed a provenance backbone – lightweight provenance model to interconnect provenance information generated by different organizations, which can be used to create an uninterrupted chain of documentation spanning the whole research process, starting from the acquisition of specimen through data generation to its final analysis and integration with other data. The chain is designed in a way that provenance pieces coming from different sources can be traversed by a unified algorithm. We have also opened and addressed practical issues such as provenance information management and inclusion and interpretation of persistent or globally unique identifiers in provenance. The opaqueness of domain-specific provenance is supported by the design of the proposed provenance model. The model is supposed to produce immutable provenance information, which can be versioned using the proposed versioning mechanism. This provides the groundwork for integrity, authenticity, and non-repudiation features.

## Methods

This section firstly describes the main three existing concepts introduced in the Related Work section in more detail and how we used them in our work. More specifically, the three concepts include the W3C PROV standard, the provenance composition pattern, and the compound activities pattern. Then we describe how the example was used to develop the provenance backbone concept and expected further work related to the backbone.

### W3C PROV

**Fig. 15:**
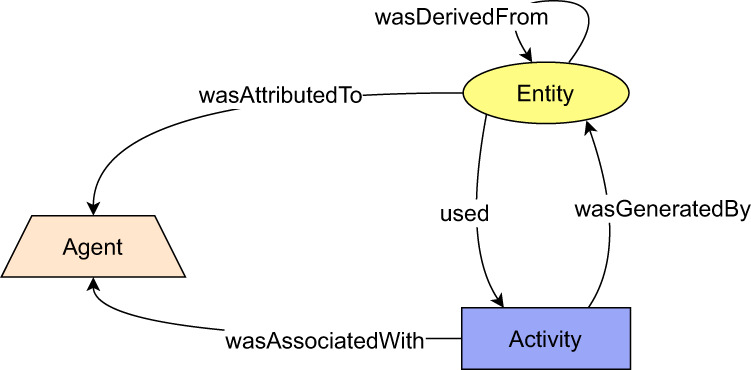
Conceptual structure of the PROV-DM (figure adapted from^64^).

PROV^21^ by W3C is a family of specifications defining the current standard for provenance information. PROV introduces a PROV-DM data model^58^ and respective serializations. Core of the model lies in provenance structures (Fig. 15) – an *entity*, an *activity* and an *agent*. An *entity* can be perceived as a snapshot of an object (which might be both physical or digital) with fixed attributes so that the same object can be expressed in provenance information by multiple entities, expressing different snapshots of the same object. *Activities* express processes that act upon objects represented by entities. *Agents* can be used to express responsibility for entities and activities. The structures can be inter-related using a pre-defined set of relations, such as *prov:wasDerivedFrom*, *prov:wasInformedBy*, or *prov:wasGeneratedBy*. Provenance structures can be packed into PROV bundles. PROV bundle is a named set of provenance statements, which is assigned an identifier so that it can be represented in provenance as an entity to express *provenance of provenance*, or simply meta-provenance. Finally, all PROV provenance structures and relations can be specialized using pre-defined extensibility points to express specialized semantics – e.g., domain-specific semantics, when applying the model in particular domain. PROV-CONSTRAINTS recommendation^48^ sets rules to generate a normalized form of resulting provenance information and defines rules to verify its validity.

The PROV data model has been used as an underlying provenance model, which we extended to define provenance backbone. More specifically, provenance structures defined in provenance backbone are extensions of the proposed PROV-DM provenance structures. The structures are listed in Table 2.

**Table 2:** Mapping between provenance backbone types and PROV-DM types.

	Provenance backbone type	PROV-DM type
1.	*senderConnector*	*prov:entity*
2.	*receiverConnector*	*prov:entity*
3.	*jumpForwardConnector*	*prov:entity*
4.	*jumpBackwardConnector*	*prov:entity*
5.	*externalInput*	*prov:entity*
6.	*mainActivity*	*prov:activity*
7.	*receiptActivity*	*prov:activity*
8.	*senderAgent*	*prov:agent*
9.	*receiverAgent*	*prov:agent*

Provenance backbone and attached domain-specific provenance can be packed into a PROV bundle, which forms an individual bundle in a provenance chain. Each PROV bundle contains connector entities to interconnect the bundles, forming a distributed chain of PROV bundles. Since each PROV bundle has been assigned an identifier (e.g., PID in an appropriate format), the bundles can be expressed in provenance to document meta-provenance. This mechanism was used to design a bundles versioning mechanism for distributed provenance chains. Since each bundle is generated during a finalization event, all the normalization and validation procedures defined in PROV-CONSTRAINS can be applied to generate finalized provenance.

### Provenance composition pattern

The provenance composition pattern is an application of the idea of shared identifiers to create interconnections between standalone PROV bundles, which document two communicating processes. The communication of two processes, a sender and a receiver, is expressed using a common entity called a message. Both sender and receiver generate a piece of provenance information for their processes and encapsulate it in their own bundle. In the sender’s provenance, the outcome of the sender’s process is expressed as an entity, and this outcome is to be sent to a receiver. The sender’s outcome is then used as an input of the receiver’s process, so the receiver includes entities that represent what has been received in his provenance information. In particular, the message entity represents a pairing between what has been sent and what has been received, and is included in both sender’s and receiver’s provenance information, and is related to respective entities using *prov:wasDerivedFrom* relation.

The *senderConnector* and *receiverConnector* present in provenance backbone can be seen as a refinement of the messages presented in the composition pattern. Both of them are based on the idea of shared identifiers and are designed to handle technical information to navigate to a consecutive provenance bundle. The main difference lies in the exact definition and usage of the structures. The messages represent a mapping between what has been sent and what has been received, but the pattern is missing a definition of how to document a time interval between sending and receiving an object. The reason for this might be that the provenance composition pattern was designed primarily for the exchange of digital messages, where the integrity of the messages can be preserved during the message transmission, so the differences between what has been sent and what has been received are not expected. In contrast, this is an important property in the context of describing the biological material transfer, which can take up to days, and unlike a dataset, the state of biological material is continuously changing. To provide direct support to describe these situations, the definition of *senderConnector* and *receiverConnector* is focused on states of described objects and better support for continuous and uninterrupted documentation of what happened. Technically, the provenance backbone suggests using *receiverConnector* to represent a *sent* object in the receiver’s provenance and a derivation relation to express what has been received. This is an explicit expression of that the sent and the received objects may be something else. The message is more similar to *jumpForwardConnector* and *jumpBackwardConnector*, which were introduced to deal with missing provenance components having an explicit meaning that a description of some intermediate steps is missing.

Finally, the provenance composition pattern does not prescribe structure of derivation paths between inputs and outputs of a documented process, so it does not provide support for navigation through distributed provenance chains using a common algorithm.

### Compound activities pattern

**Fig. 16:**
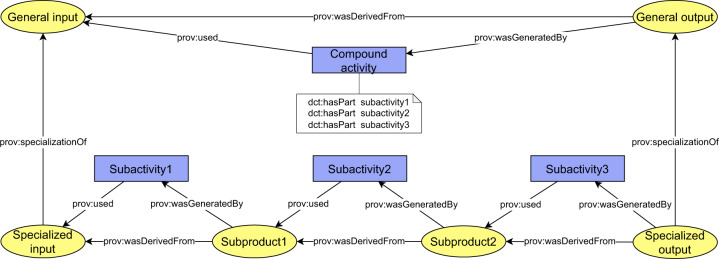
Compound activities pattern.

Compound activities pattern^23^ is a pattern for modeling sub-activities of compound activity. More technically, the pattern prescribes how to relate a compound *prov:activity* with its subactivities, which are represented as a standalone *prov:activities* in the same PROV bundle. The compound activity uses *dct:hasPart* attribute^59^ to capture list of related subactivities, of which the compound activity consists of. The subactivities then use and generate entities, which are inter-products of the main activity. The first and the last entity in the chain of entities exchanged between subactivities are a specialization of entities, which are used and generated by the main activity. The whole pattern is depicted in Fig. 16.

We suggest application of the compound activities pattern to attach domain-specific semantics to provenance backbone. In particular, the *receiptActivity* and the *mainActivity* of the backbone can be interpreted as compound activities so that the pattern can be applied. Sub-activities of the main activity and their inter-products are then considered as a domain-specific provenance interconnected with the backbone in a prescribed way.

### Methods and future work

This work started as a reaction to a gap analysis, which we conducted as a part of a standardization procedure within the ISO TC276 WG5. The proposed provenance model was developed in two main development tracks: the development with manually-generated provenance; and the real-world integration of the models into a chosen phase of the digital pathology research pipeline.

The goal of the development with manually-generated provenance was the designation of the underlying provenance model and requirements. This included iterative discussions of the authors, each contributing with knowledge from different domains related to the use case – computer science and provenance, biological material handling, data generation, and AI-based computational workflows. This way, the main author of this work was able to design and continually refine the proposed provenance model based on the feedback and suggestions from the other authors.

The goal of the development with automatically generated provenance was to show the feasibility of the provenance model by its integration with a real-world environment. Instead of implementing the automated provenance generation for the whole distributed research pipeline, we decided to create the implementation for the computational steps (septs 3b, 4, and 5) of the pipeline, which are WSI data preprocessing and AI model training. The first version of the implementation was programmed as part of a bachelor thesis^60^ during spring 2021 and focused on the expression of domain-specific semantics related to the AI workflow in terms of PROV-DM and on interconnection of resulting bundles using connectors. The current version of the implementation uses not only the connectors but the whole provenance backbone model – including the connectors, derivation paths between them, and attachment of the domain-specific semantics. The current version was implemented in early 2022 and is referenced from the Data and Code Availability sections.

Continuous results of this work were reported in the EOSC-Life project WP6 meetings, the project deliverable, and to ISO TC 276 WG5 semi-annual meetings. The proposed model was presented to internal EOSC-Life projects (https://www.eosc-life.eu/) in February 2022, with aim to be further adopted by the projects. Preliminary results were also presented to provenance community^20^, European biobanking community^61^, and European medical informatics community^62^ so far.

Our future work can be divided into two main directions. The first is aiming for rigorous validation of the proposed model in different domains, including multiple scientific communities and industry; and the second is to finish the development of support for the additional requirements – provenance opaqueness, integrity and non-repudiation, and their integration with provenance versioning mechanism, which were drafted in this work. For the purpose of rigorous validation, the model will be provided as an input for the ISO 23494 provenance standardization for domain-specific provenance information (parts 3, 4, and 5 of the standard), where it will be used to interconnect provenance coming from various areas in biotechnology (e.g., genomic data compression, computational workflow experiments, optical microscopy experiments, biological samples handling). We will also apply the proposed model within the BY-COVID project (https://by-covid.org/), which aims to develop a platform to integrate sources related to viral infections (clinical data, biological material, research results). Within this context, we can expect extensions of the model. In particular, we expect introduction of more specific types of connectors to support advanced methods for navigation through the provenance chains and more specific types of relations to attach domain-specific provenance. Another refinement may be related to set limitations on number of connectors. The reason is that some datasets are heavily used (e.g., ClinVar (https://www.ncbi.nlm.nih.gov/clinvar/), European Nucleotide Archive (https://www.ebi.ac.uk/ena/browser/home), UniProt (https://www.uniprot.org/)), so updating their provenance too often might not be feasible.

Regarding the advanced requirements, we will integrate the model with our previous work related to creation of non-repudiable provenance^55^. In particular, we will integrate the provenance backbone concept with the proposed mechanism to handle non-repudiation evidence and show how to utilize the provenance backbone model to support up-to-date distributed provenance chains with enabled integrity verification. Results of this are expected to serve as an input for the development of the ISO 23494 provenance standard, part 6 security extensions. Our preliminary results in this direction show that we will design an additional mechanism for attaching purpose-related semantics to the backbone and to meta-provenance. In this case, this would be primarily concerned with integrity verification and non-repudiation-related evidence.

## Data Availability

The dataset is available as raw files stored in Mirax MRXS format (https://openslide.org/formats/mirax/) compatible with OpenSlide library^63^. Annotations used for the evaluation stage are available as XML files compatible with ASAP (https://computationalpathologygroup.github.io/ASAP/). The dataset is pseudonymized and access can be requested via BBMRI-ERIC European Research Infrastructure by following its access policy (https://www.bbmri-eric.eu/services/access-policies); the request should be placed via BBMRI-ERIC Negotiator platform (https://negotiator.bbmri-eric.eu/) to Masaryk Memorial Cancer Institute.

## Code availability

All supplementary material, and scripts used to process the data and generate resulting provenance are publicly available at https://github.com/RationAI/crc_ml-provenance/tree/new-auto-provenance.
